# Supplementary figures and images for: Factors Associated with Prenatal Smoking Cessation Interventions among Public Health Nurses in Japan
Source: Int J Environ Res Public Health. 2020 Aug 24;17(17):6135. doi: 10.3390/ijerph17176135 (PMC7503931; doi:10.3390/ijerph17176135)

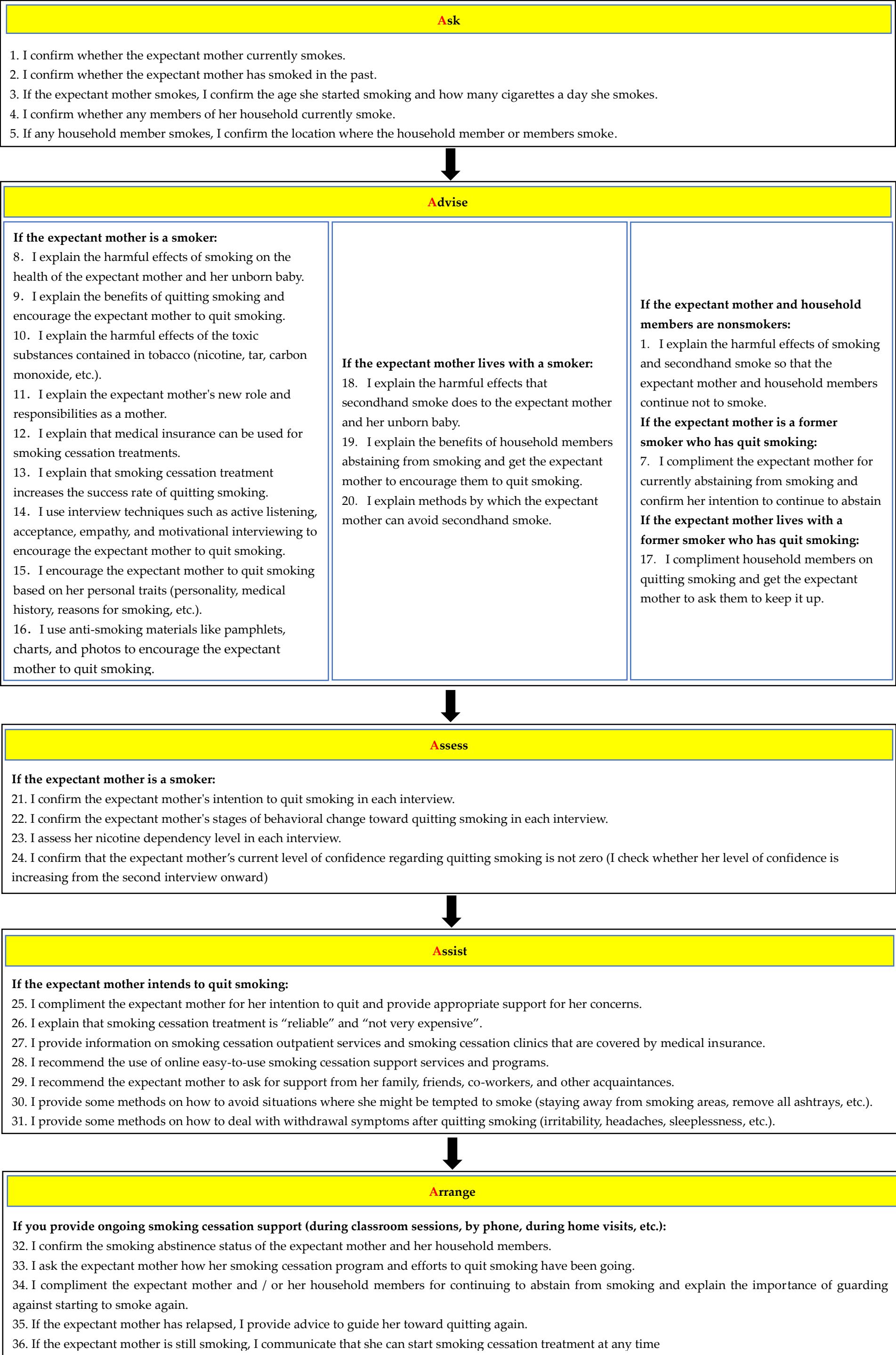

Figure 1. prenatal smoking cessation intervention draft scale.

Supplement: Supplementary file 1 [file ijerph-17-06135-s001.zip › Supplementary file 1.pdf]
